# What multiplexing means for the interpretation of functional MRI data

**DOI:** 10.3389/fnhum.2023.1134811

**Published:** 2023-04-06

**Authors:** Cheryl A. Olman

**Affiliations:** Department of Psychology, University of Minnesota, Minneapolis, MN, United States

**Keywords:** fMRI, primary visual area (V1), predictive coding, feedback–FB, neural circuits

## Abstract

Despite technology advances that have enabled routine acquisition of functional MRI data with sub-millimeter resolution, the inferences that cognitive neuroscientists must make to link fMRI data to behavior are complicated. Thus, a single dataset subjected to different analyses can be interpreted in different ways. This article presents two optical analogies that can be useful for framing fMRI analyses in a way that allows for multiple interpretations of fMRI data to be valid simultaneously without undermining each other. The first is reflection: when an object is reflected in a mirrored surface, it appears as if the reflected object is sharing space with the mirrored object, but of course it is not. This analogy can be a good guide for interpreting the fMRI signal, since even at sub-millimeter resolutions the signal is determined by a mixture of local and long-range neural computations. The second is refraction. If we view an object through a multi-faceted prism or gemstone, our view will change–sometimes dramatically–depending on our viewing angle. In the same way, interpretation of fMRI data (inference of underlying neuronal activity) can and should be different depending on the analysis approach. Rather than representing a weakness of the methodology, or the superiority of one approach over the other (for example, simple regression analysis versus multi-voxel pattern analysis), this is an expected consequence of how information is multiplexed in the neural networks of the brain: multiple streams of information are simultaneously present in each location. The fact that any one analysis typically shows only one view of the data also puts some parentheses around fMRI practitioners’ constant search for ground truth against which to compare their data. By holding our interpretations lightly and understanding that many interpretations of the data can all be true at the same time, we do a better job of preparing ourselves to appreciate, and eventually understand, the complexity of the brain and the behavior it produces.

## 1. Introduction and outline

This article has five main sections. First, a background section contains three very brief overviews that summarize some key background for this perspective and point to more in-depth review articles for readers unfamiliar with the topics. Next, a short section defines and summarizes the problem of multiplexing. Then, two sections present two different analogies–reflection and refraction–for thinking about what multiplexing means for the interpretation of fMRI data. Finally, a summary section highlights three main consequences of multiplexing: the fact that there are always multiple possible interpretations for each fMRI result does not invalidate the data; we can learn something from fMRI experiments even when they cannot be validated against ground-truth datasets and experiments; feedforward and feedback processes might be impossible to separate in our data.

### 1.1. Neural architecture

First, we will consider the neural architecture of visual pathways since examples from vision science will support key elements of our argument. Although feedforward and feedback pathways are well-defined (Felleman and van Essen, 1991; Callaway, 2004; Barone et al., 2020), it is rare to observe unidirectional information flow in the brain. Visual representations of scenes and objects do make their way up through the feedforward pathways of the early visual hierarchy to eventually reach higher visual areas and non-visual areas to guide behavior. However, at each stage, deep-layer pyramidal neurons begin to integrate/summarize the state of the local network and feed that information back to earlier stages of information processing, morphing the signal as it rises through the hierarchy. Thus, the firing rates of neurons in V1 are only predicted by retinal inputs for the first 50–80 ms (Gieselmann and Thiele, 2022). After that, we need knowledge of activity throughout the visual and non-visual networks of the brain in order to predict neural responses in even low-level sensory regions.

### 1.2. High-resolution fMRI

**FIGURE 1:**
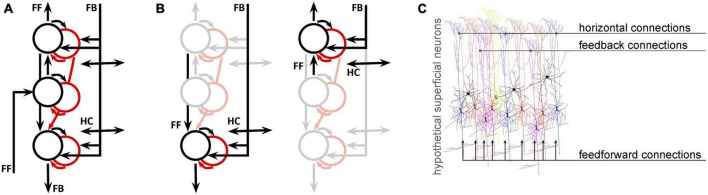
Even for fMRI data with sub-millimeter resolution, analysis and interpretation need to address the fact that multiple signals coexist at every location. **(A)** A simplified network model of deep (toward the bottom), middle, and superficial neural populations (circles: black = excitatory; red = inhibitory) and connections (arrows) in primary visual cortex. Proportions are not represented. **(B)** Copies of the first panel highlight the fact that, at the mesoscopic level (0.1–0.5 mm), neural populations receive both feedforward and feedback connections. **(C)** At the microscopic (single-neuron level) it is likely that different neurons receive different projections in different proportions. This is an entirely hypothetical illustration of superficial pyramidal neurons receiving both feedforward, horizontal (intermediate-range), and feedback (long-range) inputs, color coded according to the degree to which each neuron is modulated by each type of input. This is not intended to have specific anatomical validity, but to illustrate the likelihood that even the best-localized population has a heterogeneous feedforward/feedback balance, allowing simultaneous representation of different potential behavioral or perceptual states.

Next, we will consider the spatial scale of fMRI data compared to the neural networks we want to study. Over the past decade, there has been a notable increase in the availability of ultra high-field scanners and sub-millimeter data acquisition techniques. Advances in both acquisition (Adriany et al., 2012; Huber et al., 2017) and denoising (Vizioli et al., 2021) approaches are facilitating the wide-spread use of depth-resolved fMRI (Koopmans et al., 2010; Olman et al., 2012; Kok et al., 2016; Jia et al., 2020; de Hollander et al., 2021), which seeks to distinguish fMRI responses that are driven primarily by feedforward, intra-regional computations, or feedback based on the location of the response in the depth of cortex. While depth-resolved fMRI signals do probe neural populations that might be biased toward feedforward or feedback responses, fMRI cannot separate these pathways, which are mixed at every depth. This is not a unique limitation of the methodology–even electrophysiological or optical measures that can isolate responses of individual neurons must also wrestle with the fact that the membrane potential, spiking rate, and spiking pattern of each unit is determined by a different mixture of feedforward, lateral, and feedback signals (Figure 1).

### 1.3. Theoretical neuroscience

Finally, we want to bring into the conversation some perspectives from computational neuroscience, which considers questions about what information is represented in these neural networks, and why. Most leading theories include the idea that the computation is iterative: while different regions of the brain do produce responses that are selective for different aspects of a given task, none works in isolation and feedforward/feedback loops are pervasive (Kawato et al., 1993; Rao and Ballard, 1999; Friston, 2003; Aitchison and Lengyel, 2017). Not only does it seem as though our brains, in everyday life, use sensory data to update an existing understanding of the world (as opposed to building that understanding from scratch in each moment) but it also appears as though this updating is accomplished by feedback from higher to lower visual areas (Bastos et al., 2012). Thus, there are at least two kinds of signals simultaneously present in a given area of the brain: signals from the feedforward pathway and signals from the feedback pathway. Given the existing electrophysiological evidence for the heterogeneity of contextual modulation of neurons in primary visual cortex at each depth in cortex, it is likely that competing hypotheses are represented in spatially mixed neural populations, some receiving stronger feedforward inputs and others receiving stronger feedback inputs (Figure 1C). Thus, it is very difficult to predict–either for a single neuron or for a well-localized, 0.5 mm voxel–which computation is more strongly reflected in the mixture of field potentials and action potentials that determines the local cortical response.

## 2. Multiplexing is not a surprise

At this point, we’ve laid out the basics for an argument that there is no such thing as “the fMRI response in ___,” where you can fill in the blank with whatever brain region you are interested in understanding. Of course, there is a single response, in the sense that each voxel has a single measured intensity at a single point in time. But it can be problematic when “the fMRI response” is used to imply a singular or uniform neural response, because there is not just one neural population response in a region or a voxel. The diversity within the population is striking, and the sources of the signals that influence the local responses are far flung, such that interleaved sub-populations are simultaneously representing different sensory and behavioral states and goals and reflecting computations throughout the brain. This is the problem of multiplexing, and it will remain with us even as technology continues to improve and our resolution becomes more precise.

This problem of multiplexing is not unique to fMRI and has been considered in single-unit electrophysiology for a long time. A full understanding of a single neuron’s response requires not just measurement of the firing rate, but also the timing of specific spikes which can, for example, multiplex representation of color and pattern (McClurkin and Optican, 1996). In another example, the variance of a single neuron’s firing rate might signal uncertainty (Lengyel and Dayan, 2006). From these and other examples, it has been well established that, because multiple signals are multiplexed in even a single neuron’s spiking activity, the answer to “What information does this neuron represent?” depends on the techniques we use to ask the question.

Functional MRI researchers have long been cognizant of this problem, as well, and a large array of analysis techniques are available to address the issue on a scale appropriate for fMRI. Straight-forward univariate analyses estimate response amplitudes by regressing voxel time series against predictors derived from the experiment design and behavioral responses. Univariate analyses are still common and a valuable tool for understanding local neuronal population activity, but they are rarely considered best practice if applied in isolation. Multi-voxel pattern analyses, encoding models, and connectivity analyses are now part of the standard toolkit for fMRI analysis, and the remaining sections of this paper argues that their power and popularity come from the fact that they address the fact that neural population responses are reflected and refracted in the fMRI signal.

## 3. Reflection: Treating each fMRI response as just one node in a dynamic network

When we see a reflection in a mirror, we generally know that the object or scene that we are seeing is not actually in the mirror but is standing or happening outside the mirror. The reflection we see is made possible by the nature of the mirror–due to metallic polish or layering of dielectrics, light is turned. So we do learn something about the makeup of an object when we see a reflection in it; rarely, however, do we confuse the mirrored surface for the reflected object we are perceiving.

This section picks three lines of research addressing visual representations of information in primary visual cortex–imagery studies, contextual modulation studies, and estimation of receptive field location–to demonstrate the degree to which visual responses originating in cortical regions outside V1 are reflected in the V1 fMRI signal. In each case, the nature of the information modulating the V1 signal is such that it must originate in cortical regions outside V1, i.e., through feedback pathways. These examples are selected to illustrate instances in which cortico-cortical feedback is just another type of input to V1 and highlight the importance of analysis techniques, such as functional connectivity, that let us characterize these crucial signals driving V1 responses.

Perhaps the most straightforward situation in which the V1 fMRI response reflects neural computations that originate elsewhere in the brain is in studies of visual imagery or expectation. In these studies, a retinotopically-localized response in V1 is measured even though there is no stimulus in the corresponding region of the visual field (i.e., no retinal inputs). If scene context elsewhere in the visual field suggests structure that should be present in a blank region of the visual field, the localized V1 responses reflects the implied structure (Muckli et al., 2015; Kok et al., 2016). If attention is directed to a peripheral object and participants are asked to perform a difficult feature-discrimination task on that object, V1 responses in the fovea, where there is no stimulus, indicate the attended features in the periphery (Williams et al., 2008). Finally, when participants are trained to recall specific images, on cue, while viewing a blank screen, the V1 responses show selectivity for the location and structure of the imagined images (Breedlove et al., 2020). In each case, the identity of the stimulus that is not presented, but rather imagined or inferred, can be decoded (or recovered by the solution of an inverse encoding model, as discussed below) from the localized V1 response. The selectivity of these signals is arguably due to a combination of (1) the response properties of the local neurons (why else would feedback be targeting these specific retinotopic regions?), and (2) the visual information representations created by remote neural populations. However, in these cases, the emergent V1 fMRI response is fully determined by the observer’s behavioral goals and not by any feedforward inputs.

**FIGURE 2:**
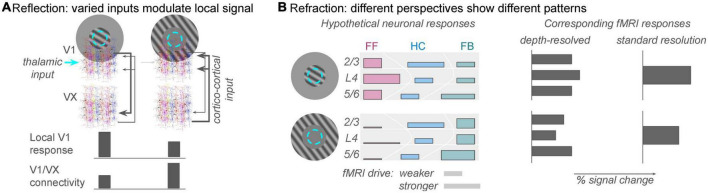
The analogies of reflection and refraction are helpful for framing population responses in V1 as a function of spatial context. **(A)** Reflection: feed-forward receptive fields in V1 are determined by thalamic inputs to the brain (cyan circle: population receptive field location), but the local V1 fMRI response can also reflect strong drive from cortical sources (gray arrows from extrastriate regions, VX). The reflection analogy refers to the fact that this non-zero fMRI response is reflecting figure/ground segmentation computations from other regions in the brain. This allows the local V1 population to perform an important comparison between new sensory information and expected scene configuration. Task-dependent connectivity analyses will discover that correlations between unmodeled fluctuations in the V1 signal and extrastriate (VX) signals are strong during the second condition. **(B)** Refraction: different analyses will show different patterns of response, but one does not invalidate the other. On the left, colored bars indicate rough predictions of responses in a population receptive field (cyan dashed circle) to stimuli that invoke different levels of feedforward and feedback inputs to the local population (approximately the size of a 3 × 3 × 3 mm fMRI voxels). Top row: stimulation of just the classical receptive field would produce dominantly feedforward responses (pinkish colors), which are strongest in the middle of the gray matter thickness. Bar height represents relative fMRI drive of neurons participating in that pathway; bar width represents estimates from recent literature of the relative fMRI response elicited by feedforward, horizontal, or feedback pathways. Bottom row: with no retinal drive in the classical receptive fields, horizontal and feedback connections will still provide input to the local population whose receptive field is stimulated. Relatively high green bars indicate feedback inputs due to strong figure/ground segmentation cues. Just as the heights and widths of the colored bars on the left are only rough summaries of the anatomical literature, there are also not yet enough data to constrain precise predictions of fMRI response from the diverse neuronal population responses. However, for the sake of argument, illustrative predictions are shown on the right. A depth-resolved analysis with sub-millimeter resolution should show reduced responses in middle and superficial depths in the absence of thalamic input, but deep layers should show relatively large responses. A standard fMRI experiment, on the other hand, should simply show reduced (but not zero) response to the surround-only stimulus.

An apparently simple but deceptively complex stimulus paradigm in vision science is orientation-tuned surround suppression. The apparent simplicity derives from its predictably: the overall response in a well-localized population of V1 neurons will be reliably suppressed when a uniform stimulus extends beyond the (shared) classical receptive field of that population (Sillito et al., 1995; Shushruth et al., 2013). If a feature contrast, such as a change in orientation, is introduced between the stimulus in the central (classical RF) region and the stimulus in the extra-classical surround region, the suppression will be reduced or eliminated. Individual neurons might actually experience response facilitation due to the feature contrast, and this is where the complexity begins to enter the picture. Several different V1-intrinsic and -extrinsic factors contribute to the suppression and release from suppression (Zipser et al., 1996; Coen-Cagli et al., 2015), individual neurons experience these effects to different degrees (Cavanaugh et al., 2002), and the magnitude of the effects depends on how the observer’s attention is directed (Roberts et al., 2007; Reynolds and Heeger, 2009). Functional MRI studies of these contrast surround suppression phenomena have produced mixed results, ranging from results that are oppose predictions from electrophysiology experiments (Schumacher and Olman, 2010), to results that show little sensitivity of the univariate fMRI response to orientation (Schallmo et al., 2016), to results that nicely match predictions from electrophysiology and behavioral measures of perceived contrast (Zenger-Landolt and Heeger, 2003). These disparate results can be reconciled if a wider range of behavior-dependent, cortico-cortical feedback signals to V1, e.g., 2nd-order contrast (Flevaris and Murray, 2015) are included among the explanatory variables that predict the V1 fMRI response magnitude. Thus, the V1 fMRI response cannot be predicted solely from firing rates measured in similar electrophysiology experiments. Accurate prediction of the V1 fMRI response requires us to include a component of the response that reflects feedback from other regions in the brain (Figure 2A).

Finally, If there is one neuroscientific claim that is broadly accepted since its first demonstration (Hubel and Wiesel, 1959), it is that neurons in primary visual cortex (V1) have spatially restricted receptive fields: a response from a given V1 neuron reliably signals the presence of an object (real or imagined) in that location. However, the vision research literature is rich with examples of the malleability of receptive fields (e.g., David et al., 2004) and we now understand even the V1 receptive field location to be an emergent property of the neural network–something that cannot be completely defined from anatomical measurements of the inputs and outputs for a neuron, but must be measured as a result of aggregated excitatory and inhibitory inputs to a neuron while the stimulus is present. The term “feedback receptive field” has recently been coined (Keller et al., 2020; Kirchberger et al., 2022) to describe the reliable way in which V1 neurons’ responses depend on cortico-cortical connections in addition to thalamocortical connections. What is important for this argument is that it is an emergent property not just of the local neural network, but of the extended (extrastriate) network. One experiment that illustrates this effect in human fMRI is the demonstration of a small but reliable spatial shift in the location of V1 responses when an object is perceived to be larger in one context compared to another (Murray et al., 2006).

Each of these examples points to the fact that V1 fMRI responses cannot be understood based solely on feedforward information and intrinsic computations mediated by lateral interactions; correct analysis of the V1 fMRI response requires additional information about inputs from other cortical regions (feedback).

This challenge is not unique to fMRI. Even electrophysiological studies that sample single-unit responses, and thus are able to characterize the temporal evolution of responses, are rarely able to determine the sources of the modulatory signals that incorporate information about context into the V1 field potentials and firing rates after the first hundred milliseconds. In sum, these raise the question of whether feedback inputs should just be considered as much a part of the V1-intrinsic response as feedforward inputs.

The challenge, of course, is that feedforward signals are much more amenable to experimental control; they can be largely determined by experimental stimuli and task instructions. Feedback signals, on the other hand, are generated by disparate brain networks that we do not fully understand, which can depend on each person’s interpretation of the scene and context that we cannot control. A powerful solution, for fMRI experiments, is offered by task-dependent connectivity analysis.

Connectivity analyses use correlations between fMRI signal fluctuations in different locations to infer neural communication or connectivity between these areas (Friston et al., 1997; Gitelman et al., 2003; Haak et al., 2012; Cisler et al., 2014). Care must be taken to exclude uninteresting sources of common fluctuation, like brain motion or imaging artifacts (Smith et al., 2013) or stimulus-evoked responses (Al-Aidroos et al., 2012). But smart analyses can rule out these confounds and use the fMRI signal to discover something about which we had no *a priori* knowledge: the degree to which signal modulation in one brain region/voxel/neural population/node in a neural network can explain or predict signal modulation in another region. Thus, connectivity analyses offer an important tool for teasing apart the intrinsic and extrinsic factors that drive a well-localized fMRI response (Schindler and Bartels, 2017).

In each of the experiments described above, performing a task-dependent connectivity analysis with the V1 response as a seed should/could/does reveal one or more other regions in the brain where residual signal (the portion of the signal that is left over after stimulus-evoked responses and physiological artifacts are removed by regression) shows task-dependent correlations with the V1 signal. Our lab is one of many that have begun including connectivity analyses in studies of V1 responses; in our case, functional connectivity has been useful for describing responses to contours embedded in complex scenes (Qiu et al., 2016; Pokorny et al., 2021). We find it difficult to draw conclusions about what these long-range correlation signals mean, but it is important to start identifying potential cortical sources of input to V1 alongside known thalamic drive. Directionality of this coupling is difficult (perhaps unwise) to determine with these correlation-based approaches. However, this ability to discover communication partners for V1 is crucial for achieving a full accounting of the inputs to V1 that determine the response, since the V1 fMRI response commonly reflects computations elsewhere in the brain that are multiplexed in the V1 signal as the visual system attempts to make sense of the visual world.

## 4. Refraction: Accepting that the fMRI signal changes appearance based on perspective

When fMRI was a relatively new technique, vision scientists with a strong background in engineering executed careful experiments designed to validate the fMRI response against neural response properties that were well-characterized by invasive electrophysiology experiments. Retinotopic mapping experiments, and then ocular dominance and orientation column mapping, validated the spatial precision of fMRI. Drawing on a strong psychophysical tradition in which behavioral sensitivity to simple image features like contrast and orientation can be correlated with the firing rates of superficial pyramidal neurons in V1 (outputs), these early validation experiments succeeded in finding strong and positive relationships between fMRI response magnitude and presumed V1 population firing rates, corroborated by perceptual reports of contrast discrimination (Boynton et al., 1999; Zenger-Landolt and Heeger, 2003).

Another series of careful experiments then started the debate about whether fMRI represents the output of a given brain region (i.e., Layer 2/3 firing rates, as in the early validation experiments) or the local computation–the mess of excitatory and inhibitory synaptic activity that integrates input, local, and feedback connections to shape the selectivity of the output responses. Comparisons between invasive electrophysiology measurements, in both animals and humans, has led to the general conclusion that local field potentials (LFPs), which measure background in flux rather than spiking of large pyramidal neurons, are the best predictors of the blood oxygenation level-dependent (BOLD) hemodynamic response (Logothetis et al., 2001; Maier et al., 2008; Hermes et al., 2019).

There is no inherent conflict between these research perspectives. Both output spiking activity and local field potentials contribute to the fMRI signal. It is likely that they do so to different degrees at different locations depending on the task and experiment design, and our current challenge is in determining the rules for those mixtures. As early pioneers, vision scientists definitely led many quantitative advancements in fMRI (Himmelberg et al., 2022). But one could also argue that vision science, with well-defined sets of external stimuli and a historical focus on recording action potentials from pyramidal cells (Olshausen and Field, 2004), has also encouraged an over-reliance on firing rates and an over-emphasis of external inputs in predicting fMRI responses. Since local field potentials reflect afferent neural activity more strongly than local firing rates (Herreras, 2016), the fact that fMRI is sensitive to both spikes and LFPs means that fMRI is an excellent tool for detecting the diverse information sources that are multiplexed in local neural networks.

Multi-voxel pattern analysis (MVPA) was first introduced as a simple linear classifier applied to visual object recognition (Haxby et al., 2001) and has grown to encompass a wide range of model-free approaches that determine whether the pattern of fMRI responses in a given region can be used to distinguish between different behavioral states or experimental manipulations. As a very open-handed approach (the classifier really is free to use any aspect of signal modulation in the included voxel population to distinguish between experimental conditions) MVPA analyses are well-suited to situations in which we cannot build an *a priori* model for exactly how the underlying neuronal population or the multiplexed signal represents a stimulus or behavioral state. Researchers who seek a principled understanding of causal relationships between task, fMRI response, and neural population activity (a “forward model”) can be frustrated by the difficulty of interpreting a positive result in MVPA analysis, but MVPA techniques are powerful tools for reliably determining whether a particular region of the brain is impacted, in some way, by an experimental manipulation (Kriegeskorte et al., 2008; Williams et al., 2008).

In contrast to MVPA, approaches that use encoding models rely more heavily on *a priori* understanding of the underlying neural responses. An encoding model is a computer simulation of what the fMRI response might be expected to look like, given our expectations for the neuronal activity in a given brain region during an experiment. Once again, vision scientists have been early adopters of this approach (Naselaris et al., 2011), but applications are certainly not limited to vision science [for examples in language and music, (Hoefle et al., 2018; Jain and Huth, 2018)]. The beauty of fMRI analyses using inverted encoding models (Brouwer and Heeger, 2011) is that one can build all sorts of neural response complexity into predictors for individual voxel responses, so the model can capture subtle differences in responses to different experimental conditions. The fact that each brain region has hundreds or thousands of voxels that can be modeled helps address the problem that the models tend to have many more degrees of freedom than the fMRI response. But the challenge, of course, is that the model can only characterize responses anticipated by the experimenter, and fMRI signal modulated along dimensions not captured by the model will go undetected (categorized as unexplained variance).

Importantly, both MVPA and encoding model approaches will produce analysis results that depend on decisions made while setting up the analysis. The results of an MVPA analysis can depend on how you construct and train a classifier, and the results of an encoding model analysis will depend on assumptions made about either the structure and function of the underlying neural responses and/or the neurohemodynamic coupling model used to translate simulated neural responses to simulated fMRI responses (e.g., the degree to which local inhibition and/or LFPs vs. spikes contribute to positive fMRI BOLD responses). As simple examples, either color or orientation (or the conjunction) can be decoded in V1 responses to stimuli that vary along both dimensions (Seymour et al., 2010), and both attended and presented direction can be decoded from population responses to moving stimuli (Kamitani and Tong, 2006). However, the potential variability in results from these analyses represents a limitation that we all need to be aware of, not an inherent flaw that negates the results of the analysis. This is where the analogy of a view through a multi-faceted prism is useful–each view provides useful information about an object that has not yet been fully described (Figure 2B). No view is sufficient to describe the object completely, but each view provides additional information about the neural responses we are trying to understand.

## 5. Discussion

The ever-increasing spatial precision of fMRI presents us with two challenges. First, the ability to pinpoint responses with sub-millimeter accuracy leads us to make claims that we have identified the location of a computation. However, if each location in the cortex is viewed as a node in an interconnected network, and if the fMRI signal reflects both local spiking responses and the sub-threshold modulatory signals measured in LFPs, then it becomes clear that localized responses reflect remote computations just as meaningfully as they reflect local computations. Second, in even the smallest fMRI voxels there are thousands of neurons, the responses of which are regulated by different mixtures of feedforward, horizontal, and feedback terms. Thus, no matter how high our resolution, multiple signals are multiplexed in each voxel such that there is not a single computation to uncover.

In the field of depth-resolved (layer) fMRI, it has been proposed that fMRI data can only be correctly interpreted in the context of a forward encoding model that relies on invasive electrophysiological measurements to provide complete characterization of the expected neural responses (Merriam and Kay, 2022). While I am entirely sympathetic with this argument and with the desire to reference experiments against solid ground truth, I also believe that this is currently an unattainable ideal. A ground truth-referenced approach requires either knowing the result of an experiment before conducting it (i.e., merely doing experiments that test whether inference of neural activity provided by non-invasive fMRI in humans produces the right answer) or possessing a validated computational model that can generate accurate predictions for laminar neural activity for novel paradigms. The latter is indeed a primary goal of computational and vision neuroscience; we seek a complete enough understanding of neural code that we can simulate realistic feedforward, lateral, and feedback neural responses. However, the field is not there yet. In the meantime, my argument is that we can continue to do fruitful, informative experiments that do update our understanding of neural responses in human brain, as long as we frame our results as pieces of the puzzle and not the whole picture.

One fundamental challenge that experimental cognitive neuroscientists will continue to wrestle with, in our continued quest to build up a more complete understanding of the brain’s function that can generalize from established paradigms to novel paradigms, is our need to study (habit of studying?) the brain out of context. True to our scientific training, we try to control experiments. Experiment control in cognitive neuroscience generally looks like asking a participant to do a specific, narrowly defined task, on cue, for a relatively short amount of time (because we need to repeat each trial several times to get reliable data). The result of careful experiment design is often that, when we isolate the elements of a task, we create disjoint and unpredictable experiences. A predictive coding framework would argue that many of the task fMRI datasets we analyze are biased toward “error” signals because of the abrupt presentation of new information. So while our careful experiment design might do an excellent job of isolating specific neural computations, which we need to do in order to understand the types of things that the brain does, it can lead us assign them the wrong priority, which makes it difficult to predict the outcomes of a new experiment with different context and task demands.

While anatomists and theorists have long known that cytoarchitectonically distinct regions of the brain are richly connected by forward and backward pathways, which are crucial to our ability to infer and learn (Friston, 2003), one of the biggest impacts fMRI has had on cognitive neuroscience has been the provision of tangible views of the interconnectivity and interdependence of brain regions, even at rest (Smith et al., 2013). Different locations in the brain are most certainly specialized for different tasks, with unique connectivity patterns that support different computations or roles in behavior [although see Westlin et al. (2023) for an argument against the “localization assumption”]. However, computations that can be detected at particular locations quite often originate elsewhere, or would not exist without both the intrinsic and extrinsic connections in a given region [e.g., V1 neurons can be described as having both feedforward and feedback receptive fields (Keller et al., 2020)]. When, for example, the widening or narrowing of a V2 population receptive field coincides with the deployment of spatial attention or an improvement in performance on a crowding task (He et al., 2019), what does it mean that the population receptive field is measured in V2? The size of the receptive field is determined both by the properties of V2 neurons and by V2-extrinsic factors, such that the size of the receptive field is both a property of the responses in V2 and of responses in other regions of the brain yet to be determined. In the end, the multiplexing of the feedforward and feedback signals requires us to draw meaning from fractured and partial views, holding multiple perspectives on neuronal function to be simultaneously true while we figure out how they fit together.

## Data availability statement

The original contributions presented in this study are included in the article/supplementary material, further inquiries can be directed to the corresponding author.

## Author contributions

The author confirms being the sole contributor of this work and has approved it for publication.
